# The value of slow-burning science: an interview with Peter Friedl and Bettina Weigelin

**DOI:** 10.1242/dmm.052037

**Published:** 2024-07-31

**Authors:** Peter Friedl, Bettina Weigelin

**Affiliations:** ^1^ Department of Medical BioSciences, Radboud University Medical Center, 6525 GA Nijmegen, the Netherlands; ^2^ Department of Preclinical Imaging and Radiopharmacy, Eberhard Karls University of Tübingen, 72076 Tübingen, Germany; ^3^ Cluster of Excellence iFIT (EXC 2180) “Image-Guided and Functionally Instructed Tumor Therapies”, University of Tübingen, 72076 Tübingen, Germany


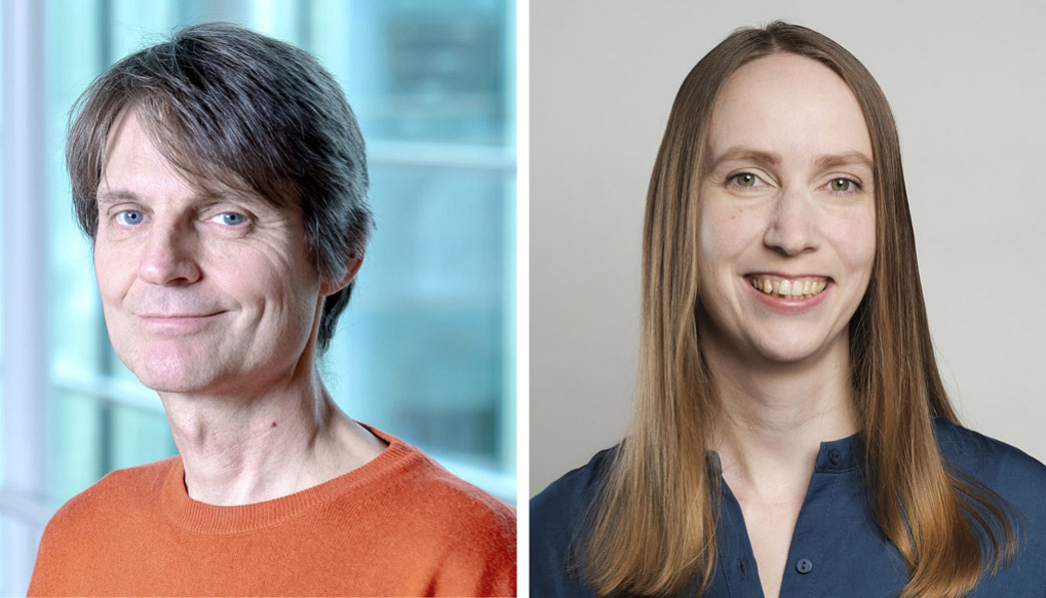


**Peter Friedl (left) and Bettina Weigelin (right)**

Peter Friedl has uncovered intricate dynamics in immune cell interactions that have had immense impact on our understanding of tumour progression and cancer therapy, with his background as a clinical dermatologist driving this research towards preclinical application and, eventually, the clinic. His laboratory is based at Radboud University Medical Centre, Nijmegen, the Netherlands, where he is also chair for the Microscopical Imaging Centre. His pioneering work with advanced microscopy gained him the European Molecular Imaging Award in 2016, and he has also received the German Cancer Award in 2008 and the Award of the Advancement of Molecular Science in 2014, among others.

Bettina Weigelin harnesses advanced microscopy techniques to investigate the mechanisms of immunotherapy, as well as cancer resistance to this therapy. She completed her PhD and postdoctoral training in Peter Friedl's group before setting up her independent laboratory at the Werner Siemens Imaging Center, part of the Department for Preclinical Imaging and Radiopharmacy, University of Tuebingen, Germany. Her impressive research trajectory has led her to receiving the New Investigator Travel Award from the Society for Thermal Medicine and being nominated for the ‘VIVA 400’ Women in Science in 2017.

While working in Peter's laboratory, Bettina discovered an unexpected phenomenon in the interactions between cytotoxic T lymphocytes and tumour cells. They observed, in a three-dimensional (3D) cell model and in a mouse model, that when these T cells interacted with solid tumour cells, the tumour cells often survived and multiple sublethal interactions with T cells would be required for tumour cell death (Weigelin et al., 2021). This phenomenon went against scientific dogma of a mandatory one-to-one pairing between cytotoxic T cells and tumour cells and required advanced developments in microscopy techniques to unequivocally prove. Therefore, this project necessitated patience and persistence, which can be challenging in an increasingly fast-paced and pressurised academic environment. Here, Peter and Bettina provide their perspectives on this research project and how this kind of valuable research can be supported in academia in the future.

**How did the project start?**

**Peter:** The project originated from an immunity paper, which we published in 2000, where we described that T cells engaged with dendritic cells to become activated (Gunzer et al., 2000). This work implied that T cells would acquire information from multiple dendritic cells until a certain threshold of activation was achieved. Four years later, this basic concept was also shown *in vivo* by the von Andrian group at Harvard, who showed that a certain type of T cell activation in the lymph node would be induced by multiple engagements with dendritic cells, followed by a more stable interaction (Mempel et al., 2004). At that point, we thought that, because we now had the tools to visualise T cell–dendritic cell interactions, we should also look at T cell interactions with tumour cells and see how the killing process happens in a 3D environment.

The first researcher to work on this in my lab was Annemiek den Boer. She generated a 3D model that would allow us to visualise the killing of individual antigenic melanoma cells by T cells that carry the specific transgenic receptor for these tumour cells. We saw quite efficient killing every once in a while but, typically, we couldn't follow T cells long enough because it was a 3D model, and they would move out of sight before killing the tumour cells. At some point, we modified the protocol and put the tumour cells on the bottom of the dish and collagen on top, so that the T cells would have to move through the collagen to get to the tumour cells. Using this technique, everything would happen in focus and we could monitor it by time-lapse microscopy, one cell–cell interaction to the next, to the next, to the next. We saw that the T cells sometimes killed reliably, but sometimes they didn't kill, and it would take multiple T cell interactions for the tumour cell to die. This is where Bettina's project started, more than 15 years ago.

**Bettina:** Indeed, I started with the task of taking over from Annemiek, who was a postdoc in the lab. She taught me in 2 weeks all the models and protocols, and at the time, she mostly worked with fibroblast cell lines, which were easy to kill through T cell interactions. She also gave me the antigenic melanoma cells, but there were not very many data with that model yet. It was when we used these real tumour cells that the project started to slow down, because we didn't see reliable killing of the tumour cells, like we did with the fibroblasts. For a very long time, I tried to understand why, by optimising the experimental conditions. That was until we started to accept that that was just the way it was, and that these sublethal interactions occur a lot more often than we expected.

**Peter:** So, we should put this into the context of where the field was at that time. It was known that T cells can kill B lymphoma cells, serially and effectively within minutes, but B cells are very antigenic and the immune synapses are very stable. However, in solid tumours, there were several studies published between 2006 and 2008 where there was no reliable killing seen in the antigenic mouse models (Boissonnas et al., 2007; Breart et al., 2008; Mrass et al., 2006), even in models of B cell lymphoma. So, there was a dichotomy between *in vitro* concepts that were already in textbooks and the *in vivo* reality. Our *in vitro* data were much closer to the *in vivo* data that didn't fit with the textbooks. At this moment, we were already in trouble, because we had to do a very solid job to turn around the perception.

I don't want to say it was easy, or there weren't phases when it was frustrating, but there was always an interesting result from the experiments that kept me going

**What motivated you to continue with this project?**

**Bettina:** Looking back, I would have liked to get the perfect dataset immediately, but I think that every PhD student goes through the phase where nothing seems to work, so it didn't feel unusual at that point. I do remember getting to the point when I was thinking that T cells probably just don't kill tumour cells at all because it was so hard to catch. But because it was so early on in my PhD, I wasn't stressed with finishing and having a complete dataset. I was free to accept that maybe what we were seeing with these sublethal interactions was real. So, I could then ask whether it biologically makes sense and think about ways of proving it and about the consequences of these interactions.

I don't want to say it was easy, or there weren't phases when it was frustrating, but there was always an interesting result from the experiments that kept me going. Also, the imaging allows you to directly see what is happening, which makes it easy to believe unexpected results. Having to establish all the different tools also kept me distracted from thinking about the possibility of failure of the project. In the end, it all came together. Then, it's fun, because you're going against the textbook a little bit. It was very rewarding.

**Peter:** I can reiterate what Bettina said, that slow progress helps. It's like being in a thick fog, where you can only see ten metres ahead, but you need to make a decision about what to do to take a step forward. There was always a little light when we took a step further; it was never total darkness. So that kept the motivation up.

As a group leader, you have to make the decision to wait or to publish what we have, despite the project not reaching full maturity. But because Bettina was a PhD student, it was important to have side projects that were productive. So, Bettina always published. She initially had smaller papers (Weigelin et al., 2012; Weigelin and Friedl, 2010) and she had a big review early on that gave her exposure (Friedl and Weigelin, 2008). Then, there was a collaboration that gave her a PNAS joint-first authorship paper (Weigelin et al., 2015), and a contribution to an important Science paper with colleagues from the USA (Denais et al., 2016). So, there was always productivity and, even without this key project, she could have graduated. So that's very important.

The third ingredient is that you need to have money. I was lucky enough to attract personalised grants that go a bit longer and are a little bit broader. That was feeding the lab. So, there was always enough money to cover this and, therefore, we had a technician who partially supported Bettina's activities.

Last, but not least, is time. In the Netherlands, a PhD project generally lasts 4 years, but Bettina's was 6 years or even longer. Then Bettina was able to continue this work as a postdoc for the revisions of the manuscript. This is something quite unusual and often hard to achieve, because the career transitions in academia force people to move on within maybe 5 years. This system goes against slow-burning projects, I must say. And so, we could keep the personal continuity in terms of Bettina being in the driver's seat throughout all these years; otherwise, I don't think it would have succeeded.

**A lot of the initial experiments focused on melanoma. I assume this concept has broad impact for other tumour types as well?**

**Bettina:** I would see no reason that this is different than other tumour types. It would probably be slightly different in each tumour type, because it depends on the general ability of the cells to repair DNA damage. But the principle remains the same.

The value of this work is that it opens the doors to general therapy concepts. And that's very gratifying to see.

**How does this concept of sublethal interactions impact our understanding of cancer therapy resistance? If subsequent contact between the T cells and tumour cells is delayed, could these sublethal ‘attacks’ induce further cancer cell mutagenesis and resistance to therapies?**

**Bettina:** Formally, we cannot say that we have shown resistance to therapies following sublethal interactions, but I think it is very easy to imagine that this will happen. Mutagenesis occurs with therapies such as sublethal chemotherapy or sublethal irradiation; so, if T cells cause DNA damage, I would assume that some mutagenesis would occur. But we have not formally shown it and we don't know to what extent it happens.

It is a valid concern that unsuccessful immunotherapy could leave a trace of additional mutagenesis. So that's a possibility. Possibly, it could be seen in patient data. But then, how do you segregate it from the background mutagenesis that a late-stage cancer has anyway? So that's going to be very difficult to disentangle.

**Peter:** This sublethal damage is not only caused by T cells when they don't kill tumour cells, but can also be induced by any type of drug. This is a new direction that we are pursuing. It could mean that a low dose of a drug, that is not cytotoxic, may cause a certain amount of stress in the target cell. This stress will be survived by the cell and could lead to an adaptation response, which we then call resistance, or it could lead to a vulnerability that makes the cell more susceptible to another stressor or another type of drug. So, we need to understand these sublethal effects, not only for immunotherapy, but for any type of therapy. The value of this work is that it opens the doors to general therapy concepts. And that's very gratifying to see.

**It seems that innovative imaging technology was essential for this project. How will these technological advances potentiate further research?**

**Bettina:** The imaging technology was certainly essential. If you don't see these inefficient interactions and you just look at the endpoint – how many cells are dead, based on how many T cells were added – you will not be able to see this phenomenon. It was also essential to develop the 3D model. It was just a minor change – we just added a collagen matrix on top of the monolayer of target cells – but it gave the T cells the task to search for the tumour cells. I think what's even more important was that once they found the tumour cells, they had a chance to decide if they stayed or if they moved on, as they do *in vivo*. We then were able to see this *in vivo*, and the intravital microscopy was essential for this.

**Peter:** We also invested many years in expressing damage sensors in the target cells. This allowed us to see if a trace is left behind after a T cell engages with a tumour cell, but the tumour cell survives. We came up with three reporters at that time to study this novel concept of sublethal damage that accumulates in the target cell. Developing these tools is where a lot of Bettina's time went initially.

**Bettina:** Surprisingly, all of the classical caspase markers that were available at the time didn't respond early enough to detect the sublethal damage, so these were not effective markers for our study. We needed to detect very early sublethal events, which meant that we needed to think a bit more creatively. For instance, we developed a nuclear damage and a DNA damage marker. However, we did not expect to actually see transient and sublethal DNA damage after cytotoxic T lymphocyte hits. I would have considered DNA damage as a point of no return before cell death. But this turned out not to be true.

**Peter:** We have now broadened the damage sensor repertoire to include sensors of reactive oxygen species, autophagy, cytoskeleton rearrangement and so on. This has created a portfolio to get a more complete view into what goes wrong in the cell and how different perturbations add up, and we call it the ‘damage fingerprint’.

These damage sensors can also be used for recording phototoxicity. When you perform microscopy on live-cell cultures, you induce energy, and this energy can cause a lot of damage in cells, perturb their function or eventually kill them. That's why microscopists are now using lower light exposure, with higher-sensitivity detectors. But sublethal damage still happens below the cytotoxic threshold. We now implement cell models that can be used for standardised detection of phototoxicity to ensure that our microscopy approach is not stressing the cells and changing the overall effect of an intervention.

**Bettina, has this project shaped your current research and how you run your own lab?**

**Bettina:** I still enjoy slow-burning projects and explaining unexpected results. The experience certainly helps me to allow these projects to evolve, because I've seen it work once. But for the slow-burning projects, you really need continuity and funding that lasts longer than usual. Otherwise, it's really difficult, especially as PhD students need to publish and present at conferences. So, I try to follow the strategy of having parallel projects that can be completed more quickly alongside a slow-burning project. I'm also at the beginning of setting up my own lab, so there are additional constraints of just getting everything started.

I think there are a lot of failed projects and failed hypotheses out there that have never been followed up but would probably lead to interesting results if somebody had the time and money to do them

**What are the persisting challenges with a slow-burning project?**

**Bettina:** With these slow-burning projects, it takes you at least a year or two to realise that the hypothesis that seems the most logical and is based on textbook knowledge is not working. When you have the first data that show this, you have to acquire more data and experience before you can trust that the hypothesis may be wrong. Only then, actually, does the project really start. Then, you can progress in a more straightforward way by deciding which tools you need and then establishing these tools and doing the experiments. So, it's this initial ‘failure’ that already takes 1 or 2 years. But in the current system, you have 2 or 3 years for a postdoc project, and if it takes you one and a half years to reassess your hypothesis, you have a problem. Then, you have to find a quick fix or stop the project because the person driving that project will have to leave before you really started. I think there are a lot of failed projects and failed hypotheses out there that have never been followed up but would probably lead to interesting results if somebody had the time and money to look into them.

**Peter:** I have perceived in science over the decades systematic dismissal of negative data – negative data being the output from a hypothesis or experiment that is not as expected. But this is where the interesting part of a slow-burning project starts and you have to explain a negative finding. However, we have a structural problem in academia, because we always have 3- to 5-year projects. If you go to a grant agency and say, ‘We had a failed experiment, but I think we can explain it if you give us more money’, you will never get money for this. So, the system doesn't allow us to understand so-called failure. And that's where we waste a lot of resources. We need to change this as a community, and our example here is just a little window into this basic problem of explaining negative data.

Certain environments can enable certain ways of research. If you have more structured security as a group leader, so you're not out after 3 years unless you find a high-profile grant, you have a little bit more time to develop. The host institution and the way they operate their business makes a big difference. I know that the Max Planck Society gives you up to 9 years of support at that stage, so that's a good environment for a slow-burning project.

We need to change this as a community, and our example here is just a little window into this basic problem of explaining negative data

**Bettina:** I was lucky to start my new lab embedded within the Cluster of Excellence ‘Image-guided and Functionally Instructed Tumor Therapies’ (iFIT), which provided funding and time for setting up the microscopes and projects. This was incredibly helpful for starting up and following up on slow-burning projects. But not everybody has that, and I think that academics need more structural support that gives you security over longer periods. Especially when starting up, so many things go wrong and, no matter how prepared you are, it takes you at least 2 years to get started, particularly when you have to build an infrastructure. So, if you have limited time to be productive, you won't have a chance.

**Peter:** For vertebrate animal research, which is where immunotherapy has to go these days, it takes extra effort to write the application for just one licence and this is more work than writing a paper or a big review. So, it slows down the project further. This also needs to be considered by decision makers when they hire somebody.

**Bettina:** Yes, and you need staff stability for this work too, particularly if you consider surgery techniques. There is no use in having a staff position for 2 years, because by the time that the person is experienced and has finished their training for the surgical work, they have to leave. That is an immense loss. We need funding for permanent positions for people with core expertise.

**Despite these challenges, why do you think these slow-burning projects are important?**

**Peter:** I think these slow-burning projects are important for revisiting drugs. We emphasise binary thinking when it comes to cancer drugs, as in, they either kill cells or they don't. But a lot happens in between, so we should try to understand the intermediate processes. For example, look at statins, which have been repurposed and do a great job in cancer therapy. And so, I think there are a lot of unrecognised treasures out there that would benefit from this exact same slow-burning way of thinking.

**What do you like to do outside of the lab?**

**Bettina:** I like to be out in nature, as I used to ride horses and I grew up with a dog. But I do have to say that in the last 4 years, all these things came a bit short. Luckily, working with microscopes is a lot of fun and very rewarding in itself. Particularly as I spend so much time at my desk, I really enjoy joining the students in the lab for imaging and playing with microscopes.

## References

[DMM052037C1] Boissonnas, A., Fetler, L., Zeelenberg, I. S., Hugues, S. and Amigorena, S. (2007). In vivo imaging of cytotoxic T cell infiltration and elimination of a solid tumor. *J. Exp. Med.* 204, 345-356. 10.1084/jem.20061890 17261634 PMC2118741

[DMM052037C2] Breart, B., Lemaître, F., Celli, S. and Bousso, P. (2008). Two-photon imaging of intratumoral CD8+ T cell cytotoxic activity during adoptive T cell therapy in mice. *J. Clin. Invest.* 118, 1390-1397. 10.1172/JCI34388 18357341 PMC2268880

[DMM052037C3] Denais, C. M., Gilbert, R. M., Isermann, P., McGregor, A. L., te Lindert, M., Weigelin, B., Davidson, P. M., Friedl, P., Wolf, K. and Lammerding, J. (2016). Nuclear envelope rupture and repair during cancer cell migration. *Science* 352, 353-358. 10.1126/science.aad7297 27013428 PMC4833568

[DMM052037C4] Friedl, P. and Weigelin, B. (2008). Interstitial leukocyte migration and immune function. *Nat. Immunol.* 9, 960-969. 10.1038/ni.f.212 18711433

[DMM052037C5] Gunzer, M., Schäfer, A., Borgmann, S., Grabbe, S., Zänker, K. S., Bröcker, E. B., Kämpgen, E. and Friedl, P. (2000). Antigen presentation in extracellular matrix: interactions of T cells with dendritic cells are dynamic, short lived, and sequential. *Immunity* 13, 323-332. 10.1016/s1074-7613(00)00032-7 11021530

[DMM052037C6] Mempel, T. R., Henrickson, S. E. and von Andrian, U. H. (2004). T-cell priming by dendritic cells in lymph nodes occurs in three distinct phases. *Nature* 427, 154-159. 10.1038/nature02238 14712275

[DMM052037C7] Mrass, P., Takano, H., Ng, L. G., Daxini, S., Lasaro, M. O., Iparraguirre, A., Cavanagh, L. L., von Andrian, U. H., Ertl, H. C. J., Haydon, P. G. et al. (2006). Random migration precedes stable target cell interactions of tumor-infiltrating T cells. *J. Exp. Med.* 203, 2749-2761. 10.1084/jem.20060710 17116735 PMC2118164

[DMM052037C8] Weigelin, B. and Friedl, P. (2010). A three-dimensional organotypic assay to measure target cell killing by cytotoxic T lymphocytes. *Biochem. Pharmacol.* 80, 2087-2091. 10.1016/j.bcp.2010.09.004 20849829

[DMM052037C9] Weigelin, B., Bakker, G.-J. and Friedl, P. (2012). Intravital third harmonic generation microscopy of collective melanoma cell invasion: Principles of interface guidance and microvesicle dynamics. *Intravital* 1, 32-43. 10.4161/intv.21223 29607252 PMC5858865

[DMM052037C10] Weigelin, B., Bolaños, E., Teijeira, A., Martinez-Forero, I., Labiano, S., Azpilikueta, A., Morales-Kastresana, A., Quetglas, J. I., Wagena, E., Sánchez-Paulete, A. R. et al. (2015). Focusing and sustaining the antitumor CTL effector killer response by agonist anti-CD137 mAb. *Proc. Natl. Acad. Sci. USA* 112, 7551-7556. 10.1073/pnas.1506357112 26034288 PMC4475992

[DMM052037C11] Weigelin, B., den Boer, A. T., Wagena, E., Broen, K., Dolstra, H., de Boer, R. J., Figdor, C. G., Textor, J. and Friedl, P. (2021). Cytotoxic T cells are able to efficiently eliminate cancer cells by additive cytotoxicity. *Nat. Commun*. 12, 5217. 10.1038/s41467-021-25282-3 34471116 PMC8410835

